# Hydroxyurea Therapy for Children With Sickle Cell Anemia in Sub‐Saharan Africa: Rationale and Design of the REACH Trial

**DOI:** 10.1002/pbc.25705

**Published:** 2015-08-14

**Authors:** Patrick T. McGann, Léon Tshilolo, Brigida Santos, George A. Tomlinson, Susan Stuber, Teresa Latham, Banu Aygun, Stephen K. Obaro, Peter Olupot‐Olupot, Thomas N. Williams, Isaac Odame, Russell E. Ware, for the REACH Investigators

**Affiliations:** ^1^ Cincinnati Children's Hospital Medical CenterCincinnatiOhio; ^2^ Centre Hospitalier MonkoleKinshasaDRC; ^3^ Hospital Pediátrico David BernardinoLuandaAngola; ^4^ University of TorontoTorontoCanada; ^5^ Cohen Children's Medical CenterNew Hyde ParkNew York; ^6^ University of Nebraska Medical CenterOmahaNebraska; ^7^ Mbale Regional Hospital Clinical Research UnitMbaleUganda; ^8^ KEMRI/Wellcome Trust Research ProgrammeKilifiKenya; ^9^ Department of MedicineImperial CollegeLondonUK; ^10^ The Hospital for Sick ChildrenTorontoCanada

**Keywords:** Africa, hydroxyurea, sickle cell anemia

## Abstract

**Background:**

Sickle cell anemia (SCA) is an inherited hematological disorder that causes a large but neglected global health burden, particularly in Africa. Hydroxyurea represents the only available disease‐modifying therapy for SCA, and has proven safety and efficacy in high‐resource countries. In sub‐Saharan Africa, there is minimal use of hydroxyurea, due to lack of data, absence of evidence‐based guidelines, and inexperience among healthcare providers.

**Procedure:**

A partnership was established between investigators in North America and sub‐Saharan Africa, to develop a prospective multicenter research protocol designed to provide data on the safety, feasibility, and benefits of hydroxyurea for children with SCA.

**Results:**

The Realizing Effectiveness Across Continents with Hydroxyurea (REACH, ClinicalTrials.gov NCT01966731) trial is a prospective, phase I/II open‐label dose escalation study of hydroxyurea that will treat a total of 600 children age 1–10 years with SCA: 150 at each of four different clinical sites within sub‐Saharan Africa (Angola, Democratic Republic of Congo, Kenya, and Uganda). The primary study endpoint will be severe hematological toxicities that occur during the fixed‐dose treatment phase. REACH has an adaptive statistical design that allows for careful assessment of toxicities to accurately identify a safe hydroxyurea dose.

**Conclusions:**

REACH will provide data that address critical gaps in knowledge for the treatment of SCA in sub‐Saharan Africa. By developing local expertise with the use of hydroxyurea and helping to establish treatment guidelines, the REACH trial results will have the potential to transform care for children with SCA in Africa. Pediatr Blood Cancer © 2015 Wiley Periodicals, Inc.

**Abbreviations**
GSCDN: Global Sickle Cell Disease Network
HbF: fetal hemoglobin
MTD: maximum tolerated dose
NHLBI: National Heart, Lung, and Blood Disease Institute
REACH: realizing effectiveness across continents with hydroxyurea
REDCap: research electronic data capture
SCA: sickle cell anemia

## INTRODUCTION

Sickle cell anemia (SCA) is among the world's most common inherited anemias, and results in significant morbidity and early mortality. SCA is most prevalent in sub‐Saharan Africa, where >250,000 affected babies are born annually, representing 1–2% of newborns in some countries.^1^, ^2^, ^3^ These astounding numbers may even underestimate the true burden of disease within sub‐Saharan Africa, as accurate birth incidence rates are unknown due to the lack of systematic newborn screening (NBS) across the continent. The impact of SCA upon child mortality in areas with high prevalence is likely under‐recognized, due to lack of NBS programs and accurate health information systems to capture the deaths of children with SCA.^4^

In North America and Europe, each of which contributes only ∼1% of the global annual sickle cell births, SCA is a medical condition with recognized impact upon both the quality and length of affected lives. Effective identification through NBS is coupled with simple early interventions (prophylactic penicillin, pneumococcal immunization) and caregiver education (fever management, spleen palpation), which together have drastically reduced the morbidity and mortality associated with SCA in developed countries.^5^, ^6^ The availability of disease‐modifying treatments (transfusions, hydroxyurea, and stem cell transplantation) in developed countries has also improved the medical outcomes of patients with SCA.^5^, ^6^, ^7^, ^8^, ^9^ In contrast, without NBS for early identification, and little access to preventive interventions or disease‐modifying therapies, most babies in Africa with SCA die of acute anemia or infection within the first years of life, often without a diagnosis.^10^, ^11^, ^12^, ^13^, ^14^ Due to limited availability and use of hydroxyurea or other proven disease‐modifying therapies, a number of herbal, traditional, and perhaps unsafe treatments and procedures are commonly used to treat SCA across Africa.

In 2010, WHO recognized SCA as a significant health problem for sub‐Saharan Africa and recommended screening and treatment programs.^2^ Several countries have begun pilot NBS programs that document the feasibility of this approach.^15^, ^16^, ^17^ Such screening programs can significantly reduce the early mortality of SCA, particularly if they are linked to penicillin and pneumococcal immunization, but will not treat the underlying disease. In fact, improved identification of SCA will increase its perceived “burden” in these countries, as children diagnosed by screening will survive early childhood and suffer severe medical complications.^18^ For this reason, treatment options must be considered as countries begin to develop national sickle cell strategies.

Hydroxyurea, an oral medication with a well‐established safety and efficacy profile in developed countries, is the most plausible therapeutic option for the majority of affected patients living in resource‐poor countries, where access and safety of other potential therapeutic options, primarily chronic blood transfusions or stem cell transplantation, are currently not affordable or realistic options. We now describe a novel partnership between investigators in North America and Africa, bolstered by industry‐supported drug donation, which led to a consensus prospective research protocol that will address the current knowledge gaps regarding the safe introduction of hydroxyurea into sub‐Saharan Africa for children with SCA.

## METHODS

### Identifying the Knowledge Gaps

Hydroxyurea is a once‐daily oral medication with over 30 years of evidence, demonstrating safety and efficacy for both adults and children with SCA.^19^ Hydroxyurea capsules are rapidly absorbed and excreted with a well‐described pharmacokinetic profile, and their contents are easily dissolved in a variety of liquids.^20^, ^21^ The mechanisms by which hydroxyurea provides benefits for patients with SCA are multi‐factorial, but the most important and easily measured benefit is fetal hemoglobin (HbF) induction, which is associated with clear laboratory and clinical benefits.^22^ Several studies in the United States and Europe have demonstrated that hydroxyurea treatment of SCA reduces the frequency and severity of many acute clinical complications and also reduces mortality for both adults and children.^23^, ^24^, ^25^, ^26^, ^27^, ^28^, ^29^, ^30^ After years of accumulated evidence, hydroxyurea has become widely accepted as a safe and effective disease‐modifying therapy for both adults and children with SCA. New guidelines from the National Heart, Lung, and Blood Institute (NHLBI) recommend that hydroxyurea be offered to all children with SCA beginning at 9 months of age, regardless of clinical symptoms.^31^

Despite the large body of evidence, hydroxyurea is rarely used in low‐resource settings, particularly in sub‐Saharan Africa, where the burden of SCA is greatest. Hydroxyurea use is limited in these settings for a number of reasons, including the following three critical knowledge gaps: (1) lack of data on the magnitude and impact of SCA in low‐resource settings; (2) absence of treatment guidelines based on prospective evidence demonstrating feasibility, safety, and benefits in low‐resource settings; and (3) inexperience regarding dosing and toxicities among healthcare providers. Additional barriers to hydroxyurea usage relate to drug availability and cost of treatment and laboratory monitoring.^32^

### Rationale for a Clinical Trial in Africa

At the 2010 Global Congress on Sickle Cell Disease in Accra, Ghana and through the Global Sickle Cell Disease Network (GSCDN, http://www.globalsicklecelldisease.org), the need for a prospective clinical trial with hydroxyurea was identified, specifically one focusing on safety data, appropriate dosing, and the feasibility of periodic visits with laboratory monitoring in limited resource settings.^33^ Hydroxyurea is known to cause mild and reversible myelosuppression with predictable cytopenias that are used to determine the maximum tolerated dose (MTD).^22^, ^23^, ^24^ In developed countries, these dose‐dependent cytopenias have not resulted in serious infections or other significant clinical events.^22^, ^23^, ^24^, ^25^, ^26^, ^27^, ^28^, ^29^ However, it is unclear whether nutritional deficiencies (such as vitamin deficiencies and severe acute or chronic malnutrition) and the unique infectious comorbidities in sub‐Saharan African children, including malaria and other parasitic diseases,^34^ helminthic infections and suboptimal vaccination against important bacterial pathogens,^12^ will exacerbate these marrow‐toxic effects of hydroxyurea. Doses commonly and safely used in children with SCA living in high‐resource settings may not be safe in this environment. A prospective research trial is needed to provide these critical data.

### Designing the Hydroxyurea Trial

The realizing effectiveness across continents with hydroxyurea (REACH, ClinicalTrials.gov identifier NCT01966731) trial was designed to answer a critical set of questions about the use of hydroxyurea for children with SCA in sub‐Saharan Africa. In this unique setting, is daily hydroxyurea treatment with monthly laboratory monitoring feasible? Will treatment be safe? Will there be clinical benefits? Can the dose be escalated and maintained with only periodic laboratory monitoring? Before hydroxyurea can be introduced widely into Africa, pilot data are needed to assess the feasibility, safety, and benefits in a limited‐resource setting.

The study design process involved multiple discussions among the international team of investigators. One critical discussion point related to the relative merits of a placebo‐controlled versus open‐label trial to determine whether hydroxyurea is safe and effective in this setting, recognizing that hydroxyurea has not been used extensively in sub‐Saharan Africa. Ultimately, a placebo‐controlled study design lacked support from both African and North American investigators. The BABY HUG study clearly demonstrated the clinical benefits of hydroxyurea compared to placebo in a young age group,^24^ so another placebo‐controlled study of hydroxyurea seemed unnecessary and potentially unethical. From the viewpoint of the African investigators, despite recognizing that local comorbidities might affect the safety of hydroxyurea, it was agreed that hydroxyurea therapy should result in similar laboratory and clinical benefits, so placebo would only deprive children of a potentially life‐saving drug.

The objectives of the REACH study therefore focus on the feasibility and safety of open‐label hydroxyurea in this patient population, while also providing preliminary data on its benefits. Beyond identifying a safe starting dose, REACH should determine whether dose escalation to MTD and sustained hydroxyurea therapy are both feasible and safe. Although a true efficacy trial was not appropriate, data should be collected with regard to the laboratory and clinical benefits of hydroxyurea treatment. In addition to these clinical objectives, an important objective of REACH is to evaluate the economic cost of providing hydroxyurea therapy (including associated monitoring) at each of the clinical sites. These economic data will be important as strategies are designed and implemented to increase access to hydroxyurea in these low‐resource countries. The short‐term goal of REACH is to obtain critical pilot data regarding the feasibility, safety, and benefits of hydroxyurea for children with SCA at four distinct research settings in Africa. Although hydroxyurea is included in the WHO Model List of Essential Medications for Children^35^ hydroxyurea remains widely unavailable or too expensive in most African settings. Based on the pilot information gained in the REACH study, the long‐term goal is to work with governments to make hydroxyurea more widely available for children with SCA in Africa.

### Selecting the Clinical Sites

**Figure 1:**
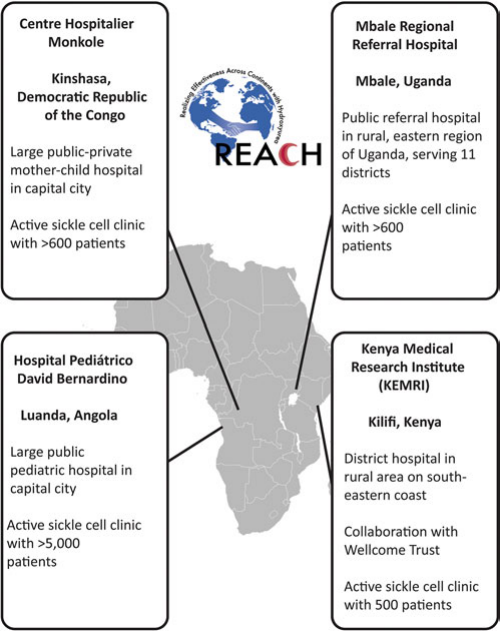
REACH Clinical Sites. After a careful screening and selection process, four unique sites across sub‐Saharan Africa with large sickle cell patient populations were selected as the clinical sites for the REACH trial.

REACH sites were selected after a careful screening process with the assistance of the GSCDN. This screening process included completion of a detailed survey requesting information about their site, which focused on their readiness to conduct high‐quality clinical research, as well as both teleconferences and site visits. Prior to official selection as a REACH clinical site, laboratory capacity was carefully evaluated, including the ability to reliably perform complete blood counts and reticulocyte counts (using an automated hematology analyzer), the ability to obtain quantitative fetal hemoglobin levels (by HPLC or capillary electrophoresis), and the ability to maintain adequate quality assurance within the laboratory. Each clinical site has one physician as the local Lead Investigator with several supporting physicians or medical officers as a part of the study team to provide clinical care for the study participants. Figure 1 provides a summary of the four selected REACH clinical sites.

### Securing the Drug Supply

The hydroxyurea drug supply for REACH will be supplied through a donation by the Bristol‐Myers Squibb (BMS) Foundation, the philanthropic arm of the large pharmaceutical company. This donation was provided through an investigator‐initiated proposal and BMS retains no oversight of the study conduct or results, except for required safety monitoring. Hydroxyurea will be supplied from the BMS commercial supply (200, 300, 400, and 500 mg capsules) and shipped directly to the clinical sites.

### Management of International Data

With multiple clinical sites across sub‐Saharan Africa, a unifying electronic data capture system was required to serve as a reliable data management system for the REACH study. The Research Electronic Data Capture (REDCap^TM^) system is a secure, web‐based data capture system used in over 70 countries. Separate REDCap environments have been developed in English, French, and Portuguese to accommodate the local languages at each clinical site.

## RESULTS

### Study Overview

REACH is a prospective, phase I/II open‐label dose escalation trial of hydroxyurea for children with confirmed SCA, between 12 months and 10 years of age, that is being conducted at four clinical sites in sub‐Saharan Africa. The sites reflect an intentional range of diversity in clinical resources, research experience, and number and types of patients, which will help the results be considered more generalizable for children with SCA across central Africa. The multicenter nature of REACH is an important component of the study design, as extrapolation of results from single center clinical trials has limitations.^36^

### Recruitment and Enrollment

The clinics at the local sites have large populations of children with SCA, with most seeing hundreds or even a thousand new patients annually. All patients will be recruited from these existing centers. The goal is to enroll and treat 150 participants at each site for a total of 600 children. Table I outlines the inclusion and exclusion criteria, which are intended to allow enrollment of most affected children, while minimizing potentially unsafe exposure of hydroxyurea to severely malnourished or chronically ill patients. The inclusion criteria do not include factors related to clinical severity, providing each site the liberty to choose those patients for whom they think hydroxyurea would provide the most benefit, regardless of clinical complications. Clinical staff will identify eligible participants and discuss the study with the patient and family in the appropriate local languages. Each site has established its own recruitment and enrollment strategies, to maintain study participants and ensure high‐quality research data collection. These strategies include the selection of families with a history of excellent compliance with clinic follow‐up, and families that live within a reasonable distance from the hospital and are willing and able to travel to the hospital for the frequent study visits.

**Table I:** REACH Inclusion and Exclusion Criteria

**Inclusion criteria**
Diagnosis of sickle cell anemia (typically HbSS confirmed by local lab techniques)
Age 1.00–9.99 years, inclusive, at time of enrollment
Weight of at least 10.0 kg at the time of enrollment
Parent/guardian willing/able to provide written informed consent
Willingness to comply with all study‐related treatments and evaluations
**Exclusion criteria**
Known medical condition making participation ill‐advised
Acute or chronic severe malnutrition as defined by WHO (weight‐for‐height or height‐for‐age Z‐score < ‐3; Appendix I)
Pre‐existing severe hematological toxicity (temporary exclusion)
Blood transfusion within 60 days before enrollment (temporary exclusion)
Hydroxyurea use within 6 months of study enrollment (temporary exclusion)

#### Study treatment

**Figure 2:**
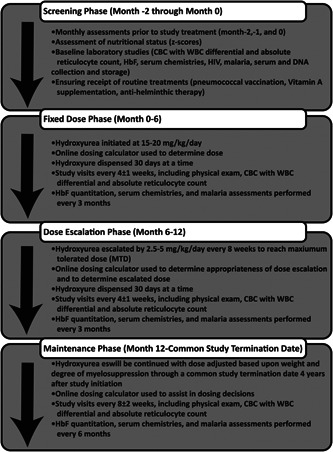
REACH Study and Treatment Phases. After obtaining informed consent, participants will proceed through each of four phases of the REACH study. Prior to initiation of hydroxyurea, there is a 2‐month screening phase, followed by 6‐month fixed dose phase, 6 months of dose escalation to maximum tolerated dose (MTD), and a maintenance phase that will continue for all participants through the Common Termination Date.

After informed, written consent is obtained, the participant will be officially enrolled in the study and begin a 2‐month evaluation period with screening studies. Once the screening period is completed, trial participants will commence open‐label hydroxyurea treatment with three phases: fixed dose (6 months), escalation to MTD (6 months), and a prolonged maintenance phase (12–36 months) until the common study termination date of 4 years from the first administered dose. Figure 2 provides a summary of the treatment phases and associated monitoring required throughout the study. Determination of hydroxyurea‐related toxicity and treatment decisions regarding hydroxyurea dosing and dose escalation will be performed using the REACH dosing calculators available on the study website (Supplementary Figure S1). The wide range of available capsule sizes allows a single daily dose of hydroxyurea for each patient.

### Primary Study Endpoint

The primary objective of REACH is to determine whether hydroxyurea therapy is safe for a large cohort of children with SCA at different clinical sites in sub‐Saharan Africa. Because each local population will be different, based on a variety of criteria such as tribal ethnicity, location, genetics, income and nutritional status, each clinical site will be considered both independently and in aggregate in analyses of study outcomes. Safety will primarily be defined by the number of participants who experience early hematological toxicity requiring temporary dose suspension or dose modification, as defined in Table II.

**Table II:** REACH Toxicity and Dose Escalation Criteria

Toxicity	Parameter	Escalation criteria	Toxicity criteria
Neutropenia	ANC (× 10^9^/L)	>4.0	<1.0
Anemia	Hb (gm/dl)	>6.5	Hb <4.0 or Hb <6.0 unless ARC >100
Reticulocytopenia	ARC (× 10^9^/L)	>150	ARC <80 unless Hb >7.0
Thrombocytopenia	Platelets (× 10^9^/L)	>150	<80

Hematological toxicities and occasional dose adjustments or medication holds are expected with hydroxyurea therapy. To account for the lower baseline hemoglobin values for children with SCA living in Africa,^37^ the thresholds for hematological toxicity are modified from those used in North American studies. Some dose adjustments will be due to drug‐related toxicity, whereas others will be sporadic cytopenias such as those occurring during infection, especially in the setting of malaria and parvovirus. For comparison, the BABY‐HUG study reported toxicities of severe neutropenia in 5% of enrolled patients, whereas severe anemia occurred in 1% and thrombocytopenia in 11%; importantly, however, hematological toxicities were also noted in the placebo‐treated arm.^25^ Similarly, the HUSOFT study reported severe neutropenia (21% of patients) and severe anemia (25%), but that study had a smaller cohort than BABY HUG, was open‐label without a control arm, and had slightly different toxicity thresholds.^26^ Based on these data, in the REACH sample size calculations, 20% of participants are expected to have a dose‐limiting hematological toxicity during the first 3 months of study treatment. However, given the potential for cytopenia that is unrelated to hydroxyurea treatment, a threshold of 30% is selected for the highest acceptable percentage with a hematological toxicity for the primary study endpoint.

### Sample Size Calculations

**Figure 3:**
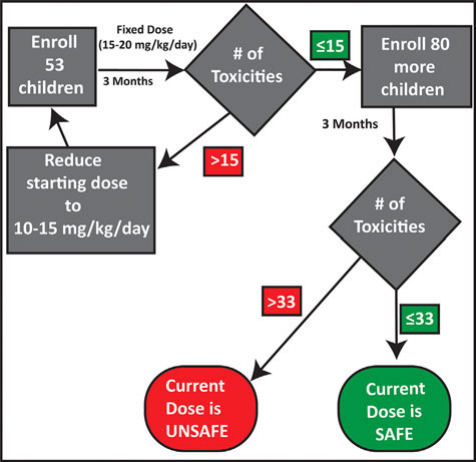
REACH Statistical Flowchart. The REACH study has an adaptive, two‐stage design allowing for early identification of hydroxyurea toxicity. The first stage will enroll 53 participants; if 15 or fewer hematological toxicities occur, study enrollment can continue to reach the final required number of 133. If there are greater that 15 hematological toxicities during this first stage, the starting dose will be reduced to 10–15 mg/kg/day and enrollment will begin again. In order to account for participant drop‐out, 60 participants will be enrolled in the first stage and 150 participants in the entire study at each site. This study algorithm will occur with independent analysis at each individual site.

Sample size was calculated using Simon's two‐stage procedure with expected and unacceptable levels of toxicity set at 20% and 30%.^38^ To achieve 90% power with a type I error rate of 10%, a sample size of 133 is required for each site (53 for the first stage), and to accommodate drop‐outs and screening failures, a total of 150 enrollments will occur at each site. Using this two‐stage approach, the first analysis will occur after 53 participants at each site have completed 3 months of hydroxyurea at the starting dose of 15–20 mg/kg/day, but will also include participants who experience a dose‐limiting hematological toxicity during the first 3 months. The main advantage of this two‐stage adaptive design is the ability to identify an unsafe drug dose early in the study, thus allowing dose modification and eventual determination of a safe hydroxyurea dose. Figure 3 outlines the statistical design and time points at which safety analyses will be performed.

### Secondary Endpoints

The safe and effective use of hydroxyurea is highly dependent on appropriate dosing and monitoring of therapy, in the setting of excellent medication adherence for all research participants. Monthly clinic visits with laboratory testing will be required during the initial phases of REACH, as this frequency of monitoring is the current standard of care in the United States. Adherence to monthly clinic visits and successful completion of required laboratory assessments will constitute the primary outcome measures of feasibility. Adherence to hydroxyurea therapy will also be determined using pill counts and the Modified Morisky Scale.^39^, ^40^

In addition to the primary safety analyses, there will be additional analyses to assess the clinical and laboratory safety of hydroxyurea for study participants. All Grade 2 or higher adverse events will be collected prospectively and evaluated carefully, particularly the infectious complications, which may be frequent and severe in this susceptible study population. The REACH study team includes a co‐investigator in infectious disease who developed guidelines for the evaluation and management of febrile events and other infectious complications. To address the lack of a comparison group or placebo‐controlled cohort, the 2‐month screening period will include three scheduled visits before study treatment initiation, with complete blood counts and collection of clinical events (e.g., fever, infections, transfusions).

Although REACH is not formally an efficacy trial, the benefits of hydroxyurea will be assessed through HbF quantitation and other hematological parameters, comparing treatment values with those at baseline and during the pre‐hydroxyurea screening period. Clinical data including growth, number of vaso‐occlusive pain crises, acute chest syndrome, infections, transfusions, and hospitalizations will also be captured as secondary outcomes to help determine treatment‐related benefits. Although pediatric sickle cell deaths are rare in high resource settings, some are likely to occur in the REACH study population. Each clinical site agreed to standardize the documentation of all deaths among their patient population (including patients enrolled and not enrolled in the REACH study) using the WHO verbal autopsy form.^41^

### Study Oversight

The REACH Data Safety and Monitoring Board (DSMB) includes individuals with extensive expertise in SCA, hydroxyurea therapy, infectious diseases, biostatistics, and global health. Additionally, an external African Advisory Board has been named, which includes national medical leaders from each African country involved in the study. The charge of the African Advisory Board will be to relay the research findings into meaningful policy within their own Ministries of Health and public health systems.

## DISCUSSION

To our knowledge, the REACH study is the first prospective multicenter clinical trial involving hydroxyurea therapy for children with SCA in sub‐Saharan Africa. The protocol was written and finalized with the full involvement and consensus of all site leaders; this approach values African site autonomy, while also providing a safe and ethical trial whose results will be meaningful. Rather than imposing North American research goals, this international partnership provides each clinical site with independence and promotes capacity building through training of site personnel in hydroxyurea use and high quality research. The research team recognized the limitations of not including a control or placebo comparison group, but felt unanimously that withholding effective treatment from patients, just for the sake of collecting comparison data, was unethical. Despite the lack of a placebo‐treated group, the significance of collecting prospective data using open‐label hydroxyurea treatment cannot be overstated, as the results could lead to wider use of the drug and integration of hydroxyurea treatment into national strategies for managing SCA. Without proper data, underutilization of hydroxyurea in this setting would likely ensue, depriving children of a potent, relatively inexpensive, and readily available disease‐modifying therapy to prevent morbidity, disability, and mortality. However, its potential overutilization or unsafe use without prospective data on proper dosing or monitoring is equally problematic; hence the need for the REACH study.

There are several unique aspects of the REACH trial that are worth noting. First is the close partnership between investigators in North America and Africa, who together wrote the final protocol after discussion and consensus. The additional partnership between academia and industry is also notable, as BMS is the main on‐label manufacturer of hydroxyurea and agreed to donate all hydroxyurea needed for this important clinical trial. Second is the intentional inclusion of four sites with geographically high burdens of SCA across sub‐Saharan Africa. Each site has a unique and diverse patient population, which will be crucial for generalizing the feasibility and safety of hydroxyurea treatment in different settings. Although the hypothesis is that hydroxyurea will be equally safe across sites, there may be important co‐morbidities within a specific population that results in increased toxicities from hydroxyurea therapy. Third is the two‐stage design of REACH, which allows for early identification of safety concerns and lowers the hydroxyurea dosing if the initial fixed dose of 15–20 mg/kg/day results in an unacceptable frequency of toxicities. Next is the internationally accessible internet‐based electronic data capture system (REDCap), which will allow the timely entry of study data in several different languages; the study website has been equipped with software tools in three languages that allow easy and accurate hydroxyurea dosing using web‐based calculators. Finally, the REACH study has expert and transparent oversight; in addition to co‐investigators who bring valued expertise, the multi‐disciplinary and international DSMB will provide expertise in safety and data review, whereas the African Advisory Board will help translate the research findings into clinical practice within each individual country.

As Ministries of Health across sub‐Saharan Africa begin to address the large and growing burden of SCA within their individual countries, the expense of such care must be addressed. The cost of quality sickle cell care in an African setting has been previously considered,^42^ but this analysis did not include costs of hydroxyurea treatment and monitoring. Hydroxyurea, a relatively inexpensive medicine (<$1 USD per generic 500 mg capsule), has the potential to dramatically reduce the rising burden of SCA in sub‐Saharan Africa by reducing the frequency and severity of acute complications (pain, acute anemia, neurologic complications) of SCA. REACH will provide an opportunity to examine the costs and benefits of hydroxyurea in four unique African settings, with the expectation that the costs of drug and monitoring will be outweighed by savings in hospitalizations and treatment of clinical complications.

Results from the prospective multicenter REACH trial could be transformative, by providing vital data that can inform the safe and effective introduction of hydroxyurea into different regions of Africa. The study also will provide critical baseline data and strengthen a Central African research consortium, which should allow more definitive follow‐up studies investigating the efficacy of hydroxyurea for specific clinical complications. The REACH trial will have even further significance by providing training about the use of hydroxyurea within the framework of local practice, thereby providing the local capacity necessary to expand the safe use of hydroxyurea in the future.

## Supporting information

Additional supporting information may be found in the online version of this article at the publisher's web‐site.

**Figure 1**. REACH Online Dosing Calculators.

Supplementary Appendix
