# Supplementary material for: Hydroxyurea Therapy for Children With Sickle Cell Anemia in Sub‐Saharan Africa: Rationale and Design of the REACH Trial
Source: Pediatr Blood Cancer. 2015 Aug 14;63(1):98–104. doi: 10.1002/pbc.25705 (PMC4825070; doi:10.1002/pbc.25705)
Supplement: Supplementary file 1 — Figure 1. REACH Online Dosing Calculators. [file PBC-63-98-s001.pdf]

A

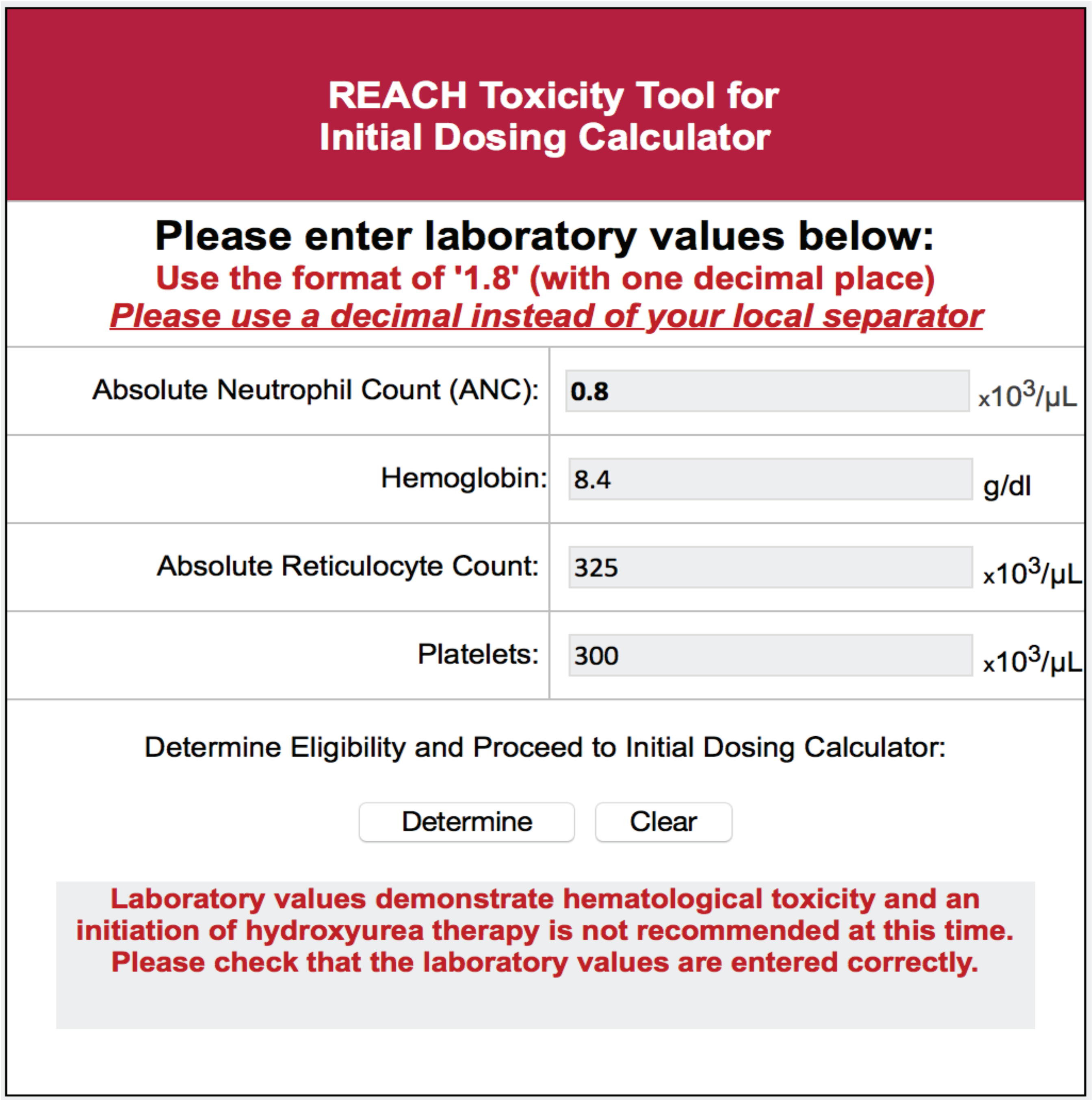

REACH Toxicity Tool for
Initial Dosing Calculator

Please enter laboratory values below:

Use the format of '1.8' (with one decimal place)

Please use a decimal instead of your local separator

Absolute Neutrophil Count (ANC):	**0.8**	$x10^3/\mu L$
Hemoglobin:	8.4	g/dl
Absolute Reticulocyte Count:	325	$x10^3/\mu L$
Platelets:	300	$x10^3/\mu L$

Determine Eligibility and Proceed to Initial Dosing Calculator:

Determine Clear

Laboratory values demonstrate hematological toxicity and an initiation of hydroxyurea therapy is not recommended at this time. Please check that the laboratory values are entered correctly.

B

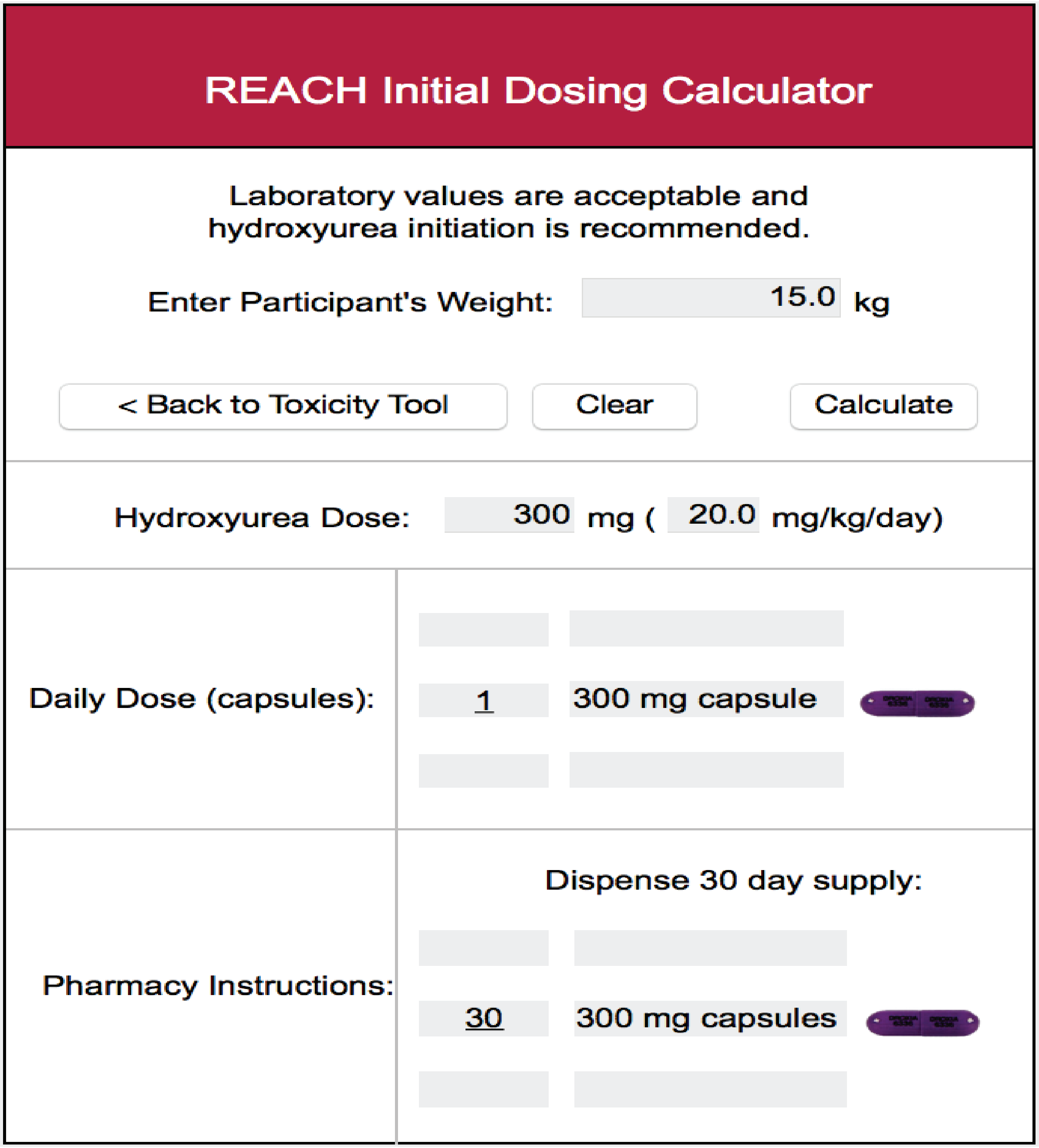
REACH Initial Dosing Calculator
Laboratory values are acceptable and hydroxyurea initiation is recommended.
Enter Participant's Weight:
15.0
kg
< Back to Toxicity Tool
Clear
Calculate
Hydroxyurea Dose:
300
mg (
20.0
mg/kg/day)
Daily Dose (capsules):
1
300 mg capsule
Pharmacy Instructions:
Dispense 30 day supply:
30
300 mg capsules

C

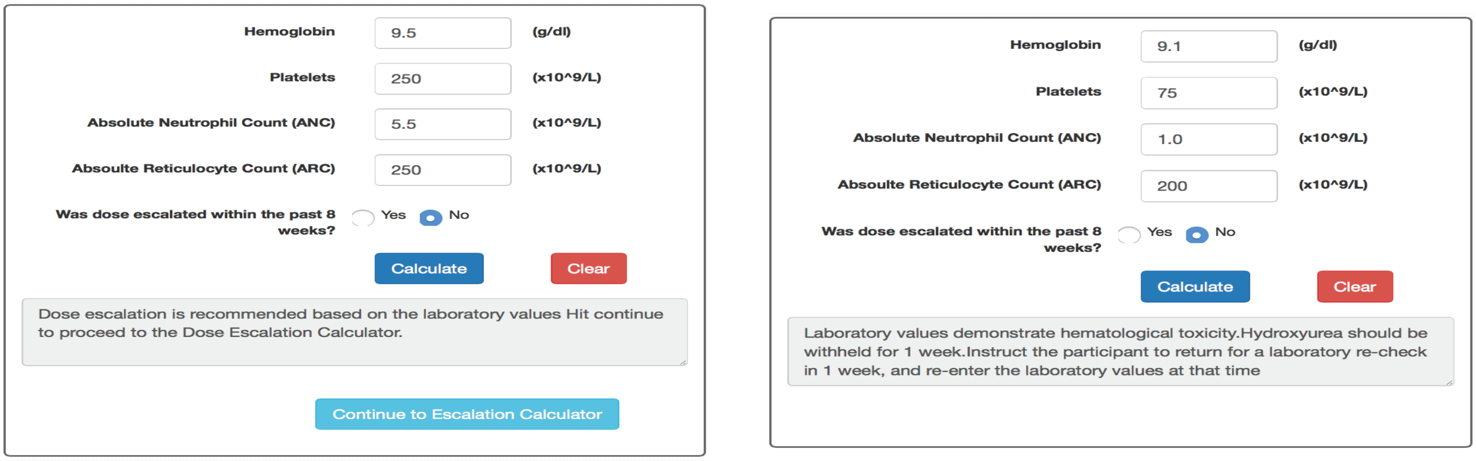
Hemoglobin
9.5
(g/dl)
Platelets
250
(x10^9/L)
Absolute Neutrophil Count (ANC)
5.5
(x10^9/L)
Absoulte Reticulocyte Count (ARC)
250
(x10^9/L)
Was dose escalated within the past 8 weeks?
Yes
No
Calculate
Clear
Dose escalation is recommended based on the laboratory values Hit continue to proceed to the Dose Escalation Calculator.
Continue to Escalation Calculator
Hemoglobin
9.1
(g/dl)
Platelets
75
(x10^9/L)
Absolute Neutrophil Count (ANC)
1.0
(x10^9/L)
Absoulte Reticulocyte Count (ARC)
200
(x10^9/L)
Was dose escalated within the past 8 weeks?
Yes
No
Calculate
Clear
Laboratory values demonstrate hematological toxicity.Hydroxyurea should be withheld for 1 week.Instruct the participant to return for a laboratory re-check in 1 week, and re-enter the laboratory values at that time

D

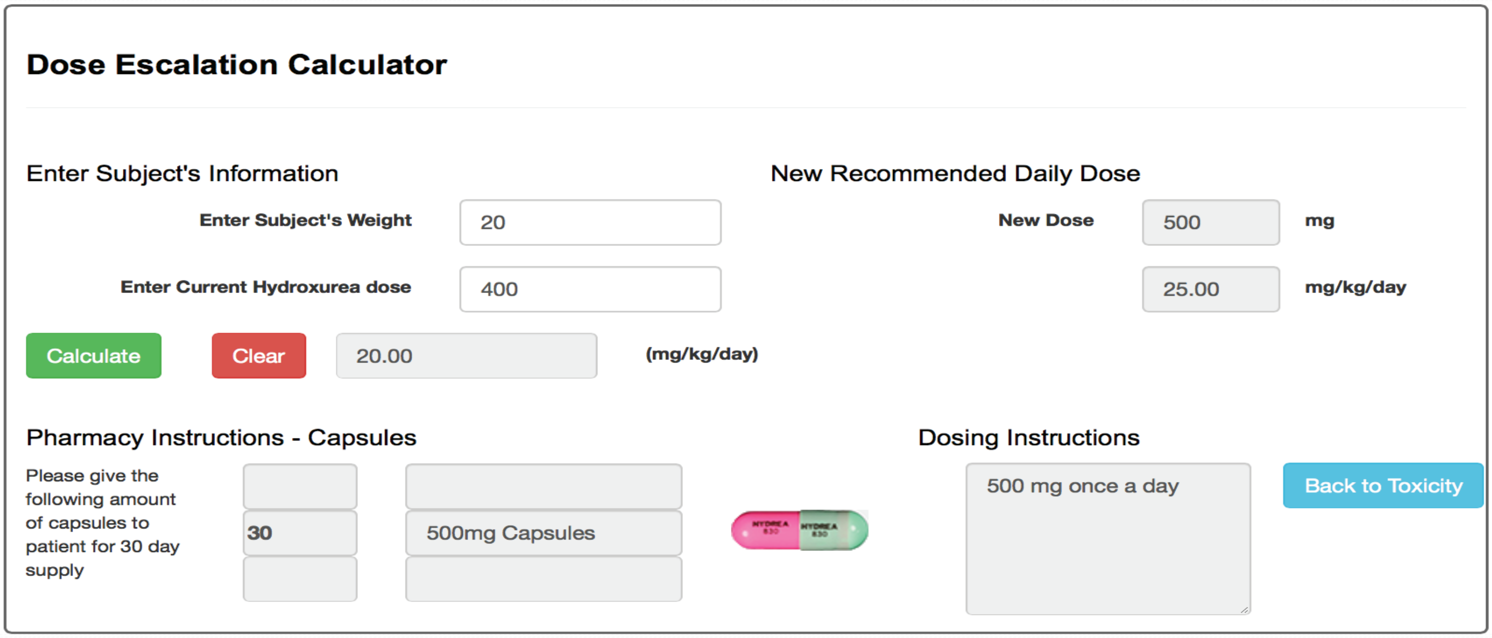
Dose Escalation Calculator
Enter Subject's Information
Enter Subject's Weight
20
Enter Current Hydroxurea dose
400
Calculate
Clear
20.00
(mg/kg/day)
New Recommended Daily Dose
New Dose
500
mg
25.00
mg/kg/day
Pharmacy Instructions - Capsules
Please give the following amount of capsules to patient for 30 day supply
30
500mg Capsules
Dosing Instructions
500 mg once a day
Back to Toxicity
