# Supplementary material for: Hydroxyurea Therapy for Children With Sickle Cell Anemia in Sub‐Saharan Africa: Rationale and Design of the REACH Trial
Source: Pediatr Blood Cancer. 2015 Aug 14;63(1):98–104. doi: 10.1002/pbc.25705 (PMC4825070; doi:10.1002/pbc.25705)
Supplement: Supplementary file 2 — Supplementary Appendix [file PBC-63-98-s002.docx]

**Supplementary Appendix I: REACH Investigators**

**Cincinnati Children’s Hospital Medical Center (Cincinnati, OH, USA):** John Boesing, Arielle Hernandez, Thad Howard, Denise Lagory, Justin McAdams, Patrick McGann, Sophie Perier, Susan Stuber, Russell Ware, Matt Wollman

**Hospital Pediátrico David Bernardino (Luanda, Angola)**: Dário André, Rosa Calembe, Jose Luis Reis da Fonseca, Violeta Chimuco, Lourenço Nassesa, Vysolela de Oliviera, Brigida Santos

**Centre Hospitalier Monkole (Kinshasa, Democratic Republic of Congo):** Léon Tshilolo, Gisèle Kazadi, Bobo Kitenge, Nancy Madingo, Jordy Mafema, Didier Mbuyi, Jacques Muhindo, Landry Kipepe, Frank Nzengu

**KEMRI/Wellcome Trust Research Programme (Kilifi, Kenya)**: Jacob Golijo, Emmanuel Mabibo, Alex Macharia, Kathryn Maitland, Vicki Marsh, George Mochamah, Neema Mturi, Ruth Murabu, Gideon Nyutu, Anisa Omar, Sabah Omar, Norbert Peshu, Jimmy Shangala, Benjamin Tsofa, Thomas N. Williams

**Mbale Regional Hospital Clinical Research Unit (Mbale, Uganda)**: Florence Masambu, Peter-Olupot-Olupot, Julius Nteziyaremye, Felix Opio, Alex Sande, Ham Wabwire

**Cohen Children’s Medical Center (New Hyde Park, NY, USA):** Banu Aygun

**University of Nebraska (Omaha, Nebraska, USA):** Stephen Obaro

**University of Toronto (Toronto, Canada):** George Tomlinson
